# Role and significance of c-KIT receptor tyrosine kinase in cancer: A review

**DOI:** 10.17305/bjbms.2021.7399

**Published:** 2022-04-27

**Authors:** Emana Sheikh, Tony Tran, Semir Vranic, Arkene Levy, R. Daniel Bonfil

**Affiliations:** 1 OMS-III, Dr. Kiran C. Patel College of Osteopathic Medicine, Nova Southeastern University, Fort Lauderdale, Florida, United States; 2 Department of Basic Medical Sciences, College of Medicine, QU Health, Qatar University, Doha, Qatar; 3 Department of Medical Education, Dr. Kiran C. Patel College of Allopathic Medicine, Nova Southeastern University, Fort Lauderdale, Florida, United States

**Keywords:** c-KIT, CD117, cancer, receptor tyrosine kinases, stem cell factor receptor

## Abstract

c-*kit* is a classical proto-oncogene that encodes a receptor tyrosine kinase (RTK) that responds to stem cell factor (SCF). C-KIT signaling is a critical regulator of cell proliferation, survival, and migration and is implicated in several physiological processes, including pigmentation, hematopoiesis, and gut movement. Accumulating evidence suggests that dysregulated c-KIT function, caused by either overexpression or mutations in c-*kit*, promotes tumor development and progression in various human cancers. In this review, we discuss the most important structural and biological features of c-KIT, as well as insights into the activation of intracellular signaling pathways following SCF binding to this RTK. We then illustrate how different c-*kit* alterations are associated with specific human cancers and describe recent studies that highlight the contribution of c-KIT to cancer stemness, epithelial-mesenchymal transition and progression to metastatic disease in different experimental models. The impact of tyrosine kinase inhibitors in treating c-KIT-positive tumors and limitations due to their propensity to develop drug resistance are summarized. Finally, we appraise the potential of novel therapeutic approaches targeting c-KIT more selectively while minimizing toxicity to normal tissue.

## INTRODUCTION

About two-thirds of the 90 tyrosine kinase (TK) genes described in the human genome encode for receptor tyrosine kinases (RTKs) [1]. These cell-surface receptors transduce a response on binding to a ligand and are defined by an extracellular (EC) ligand-binding domain, a single transmembrane (TM) region, a juxtamembrane (JM) region, a cytoplasmic portion with a conserved protein TK domain, and a flexible carboxy (C)-terminal tail [2,3]. RTKs are ubiquitously spread in multicellular animals, from the oldest metazoan phylum existing today (Porifera) to Chordata [4]. In humans, the 58 RTKs described so far are classified into 20 subfamilies or classes based on the structure of their amino (N)-terminal ligand binding ectodomains, which consist of one or more defined motifs including cysteine-rich regions, fibronectin type III-like domains, immunoglobulin (Ig)-like domains, kringle-like domains, epidermal growth factor-like domains, cadherin-like domains, discoidin-like domains, and leucine-rich regions [1,5]. Among the different classes of human RTKs described to date, Class III RTKs, which are characterized by the presence of five Ig-like EC domains, include platelet-derived growth factor α and β receptors (PDGFR α/β), colony-stimulating factor 1 receptor, fms-like RTK 3, and c-KIT [6,7]. These RTKs play a pivotal role in several aspects of normal cell physiology, and different mutations that affect them can cause aberrant downstream signaling that is often linked to many disorders, including cancer [8,9].

## STRUCTURE AND BIOLOGICAL FUNCTIONS OF C-KIT

**FIGURE 1:**
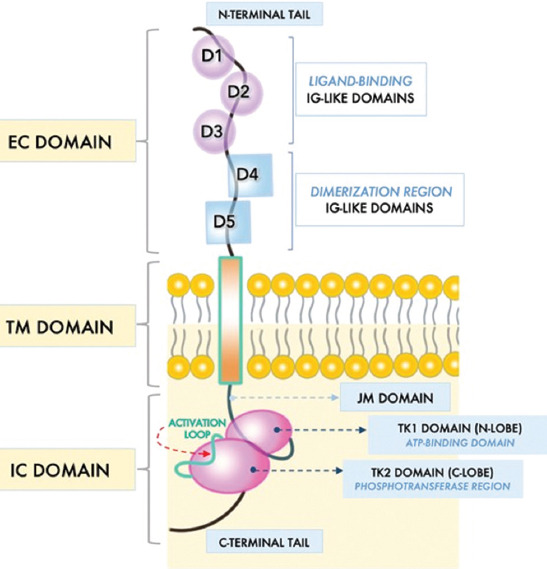
Structural organization of the human c-KIT receptor. In its inactivated state, c-KIT is present as a monomer that comprises extracellular (EC), transmembrane (TM) and intracellular (IC) domains. The outer immunoglobulin-like (Ig-like) domains D1 to D3 are key components for binding to stem cell factor (SCF), whereas D4 and D5 are essential for homotypic contacts needed for KIT dimerization. The IC domain contains a juxtamembrane (JM) domain, a tyrosine kinase (TK) domain and a flexible carboxy-terminal (C-terminal) tail. The JM domain contributes to the relay of IC downstream signaling. The TK domain is further divided into the amino-terminal TK1 (N-lobe) domain, which houses an ATP-binding region, and the carboxy-terminal TK2 (C-lobe) domain, which encompasses a phosphotransferase region and activation loop.

The proto-oncogene c-*kit*, mapped to chromosome 4q11-12 in humans [2] and chromosome 5 (W locus) in mice [10], was discovered in 1986 as the cellular homolog of the transforming viral oncogene v-*kit* in the Hardy-Zuckerman 4 feline sarcoma virus [11]. Wild-type c-*kit* encodes for a 145 kDa, 976 amino acid type IIIa RTK protein known as c-KIT, which is often referred to as CD117 or stem cell factor (SCF) receptor due to its association with its ligand SCF [12]. The c-KIT protein resides in the cell membrane and is comprised of EC, TM, and intracellular (IC) regions (Figure 1). Like all class III RTKs, the EC portion of c-KIT comprises five Ig-like domains (D1-D5). The first three domains are essential for c-KIT binding to SCF, whereas D4 and D5 are involved in dimerizing adjacent c-KIT monomers [13,14]. The EC region is followed by a single spanning TM helix that connects with the IC domain, including a JM domain coupled to a TK domain and a C-terminal tail region. The JM domain is essential for c-KIT receptor control and modulation, particularly in the relay of IC downstream signaling [15]. The TK domain is split into the proximal amino-terminal lobe (N-lobe) TK1 with an ATP-binding region, and a distal carboxy-terminal lobe (C-lobe) TK2 with a phosphotransferase domain [16] (Figure 1).

Different c-KIT isoforms generated by alternative mRNA splicing have been described, including two that differ by the presence or absence of the tetrapeptide sequence glycine-asparagine-asparagine-lysine (GNNK) in the EC domain [17-19]. Although both c-KIT isoforms have binding affinity to SCF, the GNNK-negative isoform leads to faster phosphorylation of the receptor, a more robust downstream signaling, and higher tumorigenic potential in mice [20-23]. Another c-KIT isoform results from losing one of the two serine residues in the kinase insert (KI) domain [17]. In contrast, a fourth isoform is caused by a shorter transcript of c-*Kit* that encodes a truncated c-KIT without kinase activity and only contains TK2 and the C-terminal tail region [24].

c-KIT is expressed by various cells in the body, and signaling pathways stimulated by its activation by SCF under physiologic conditions are implicated in regulating cellular processes such as cell proliferation, survival and migration [13]. In the normal bone marrow, c-KIT is expressed by hematopoietic stem cells, playing an important role in self-renewal and differentiation into various blood cells (reviewed in [25]). Indeed, homozygous white-spotted (W) loss-of-function mutations in the c-*Kit* gene (c-*Kit*^W/W^) in mice have shown to cause lethal anemia caused by hematopoietic stem cell defects [26,27]. c-KIT expression is gradually lost during hematopoietic differentiation and only retained or increased in mast cells, natural killer (NK) cells and dendritic cells (DCs), suggesting an essential function in inflammation and immunity [28,29]. Moreover, different studies have shown that CD117/c-KIT is not only expressed by bone marrow-derived stem cells, but also by those found in other organs in adults, such as prostate [30], liver [31] and heart [32], suggesting that SCF/c-KIT signaling pathways may contribute to stemness in some organs. Furthermore, c-KIT has been linked to many different biological processes in other cell types. For instance, c-KIT signaling has been shown to regulate oogenesis, folliculogenesis and spermatogenesis, exerting critical functions in female and male fertility [33,34].

c-KIT is also critical to the proliferation, survival and migration of melanocytes from the neural crest to the dermis [35]. Loss-of-function mutations in c-*kit* can cause piebaldism, an autosomal dominant disorder characterized by congenital absence of melanocytes in patches of skin and hair, similar to the “dominant white spotting” observed in mice with mutations in the same gene [36,37]. In individuals with the piebald trait, constipation is often seen because of the loss of interstitial cells of Cajal (ICC), which are c-KIT-positive cells that control gut peristalsis [36]. The role of c-KIT in ICC development is supported by studies in mice with loss-of-function mutations in c-*Kit* that present a constipation phenotype [38,39].

## ACTIVATION AND DOWNSTREAM SIGNALING OF C-KIT

**FIGURE 2:**
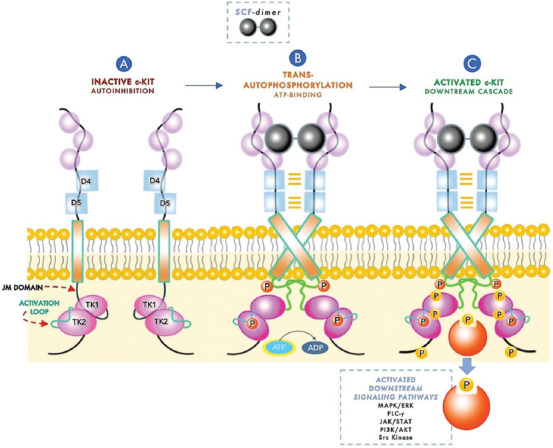
Schematic representation of c-KIT activation and downstream signaling. (A) Here, two adjacent monomeric c-KIT molecules are cis-autoinhibited by the juxtamembrane (JM) domain that inserts between the tyrosine kinase (TK) 1 and 2 domains, leading to a static configuration that sterically blocks the activation loop (AL) residing in the catalytic cleft between the lobes; (B) c-KIT is activated on binding of dimeric stem cell factor (SCF) to immunoglobulin-like (Ig-like) domains D1 to D3, which induces receptor reorientation and homotypic interaction between adjacent D4 and D5 domains. Transphosphorylation of tyrosine residues in the JM domain enables its dissociation from the TK1 domain. TK1 and TK2 domains are displaced away, allowing access to the catalytic cleft for ATP binding and release of ADP from the binding site for a second transphosphorylation of tyrosine residues in the AL; (C) Further phosphorylation of tyrosine residues in the kinase insert region and C-terminal tail creates docking sites for several substrates, leading to downstream signaling through the mitogen-activated protein kinase/extracellular signal-regulated kinase (MAPK/ERK), phosphatidylinositol 3-kinase/protein kinase B (PI3K/AKT), phospholipase C-γ (PLC-γ), Janus kinase/signal transducer and activator of transcription (JAK/STAT), and Src kinase pathways.

Under physiological conditions and when it is not bound to SCF, c-KIT resides in the cell membrane as a monomer. In this resting state, c-KIT is cis-autoinhibited by the JM domain that inserts between the TK1 (N-lobe) and TK2 (C-lobe) domains. This leads to a static configuration that sterically blocks the “activation loop” that resides in the catalytic cleft between the lobes from assuming an extended and active conformation (“JM autoinhibition”) [15,40,41] (Figure 2A). The binding of dimeric SCF to D1-D3 regions bridges two adjacent c-KIT molecules together and leads to a D4 and D5 reorientation that results in c-KIT homodimerization [42,43]. This conformational change leads to trans-autophosphorylation of selected tyrosine residues (Y) events that appear to occur in a specific order. The initial autophosphorylation occurs in tyrosine residues in the JM domain (primarily Y568 and Y570), resulting in its displacement from the N-lobe and swinging away of the loops, allowing access to ATP and release of ADP from the active site [5,13,15,16]. Subsequent transphosphorylation occurs in the activation loop (Y823) [13,15,16] (Figure 2B). Full activation of c-KIT occurs when additional tyrosine residues in the KI region (Y703, 721, and 730) and the C-terminal tail (Y900 and Y936) are phosphorylated [13,15,16] (Figure 2C).

Many of the tyrosine residues mentioned above serve as substrate docking sites after transphosphorylation, activating downstream transduction pathways that lead to various cellular responses (Figure 2C). It is important to note that the transduced signaling pathways and their consequential effects are dependent on the specific tyrosine residue phosphorylated. Here, we will provide a simplified explanation of signaling pathways downstream of c-KIT that, rather than being just linear and independent, are now known to be much more complex and occasionally connected with other downstream signaling molecules activated by other receptors.

Signaling cascades activated downstream of c-KIT include mitogen-activated protein kinase/EC signal-regulated kinase (MAPK/ERK), phosphatidylinositol 3-kinase/protein kinase B (PI3K/AKT), C-γ (PLC-γ), Janus kinase/signal transducer and activator of transcription (JAK/STAT), and Src kinase pathways [44]. On phosphorylation, Y703 and Y936 bind the SH2 domain of the adaptor protein growth factor receptor-bound protein 2 (Grb2), ending in the activation of the MAPK/ERK pathway, which plays essential roles in the regulation of gene transcription and cell proliferation [45,46]. Conversely, phosphorylation of c-KIT at Y721 triggers the PI3K/AKT pathway that promotes cell survival and evasion of apoptosis, either through direct binding of the p85 subunit of PI3K or indirectly through binding of PI3K to the scaffolding protein Grb2-associated binding protein (Gab2) and Grb2 [47-49]. The PI3K/AKT pathway can also be activated by phosphorylated Tyr900 in the C-lobe through binding to the adaptor protein Crk [41]. The PLC-γ pathway, which promotes cellular proliferation and suppresses apoptosis through the actions of diacylglycerol and inositol 1.4,5-triphosphate, can be triggered when PLC-γ interacts with phosphorylated Y730 [13,50]. In addition, activation of the Src family of tyrosine kinases (SFK) has been reported to occur through the interaction of its SH2 domain with phosphorylated Y568, Y570, and Y936, stimulating cell proliferation and survival through Akt phosphorylation and, presumably, cell migration through phosphorylation of focal adhesion kinase [46,51]. Moreover, SFK and PI3K/AKT participate in the activation of the JAK/STAT pathway [46,52], suggesting that phosphorylation of some c-KIT tyrosine residues leads to activation and translocation of STAT proteins into the nucleus, where they act on target gene promoters [53,54].

Different regulatory mechanisms function as negative-feedback loops to ensure tight control of signaling output once the c-KIT receptor is activated [55]. The main mechanisms of attenuation of c-KIT signaling include (1) c-KIT ubiquitination and internalization, (2) dephosphorylation, and (3) PKC-dependent serine phosphorylation. In the first of these mechanisms, activated c-KIT is transported from the cell surface to the interior of the cell through clathrin-mediated endocytosis [56]. E3 ubiquitin-protein ligase c-Cbl (named after Casitas B-lineage Lymphoma) binds directly to activated c-KIT receptors through Y568 and Y936 or indirectly through Grb2 to Y703 and Y936, or via the p85 subunit of PI3K [13,57,58]. Binding of another E3 ubiquitin ligase complex containing suppressor of cytokine signaling (SOCS) 1 and 6 isoforms to Y568 in activated c-KIT has also been described [59,60]. The internalized c-KIT is then targeted for lysosomal and proteasomal degradation [61,62]. Furthermore, attenuation of c-KIT signaling can occur by the action of phosphatases such as Src homology region 2 domain-containing phosphatase-1, which has been shown to associate with activated c-KIT causing its dephosphorylation [63,64]. Finally, increased PKC activation downstream from c-KIT can lead to negative feedback regulation of the receptor by phosphorylating Ser741 and Ser746 residues in the KI domain [65,66]. Moreover, PKC activation has also been shown to cause shedding of the EC domain of c-KIT, making it unresponsive to SCF stimulation [67].

## C-KIT AND CANCER

Genomic profiling of nearly 19,000 de-identified samples has shown c-*kit* alterations in 2.86% of 59 major cancer types studied, with some of them presenting very frequent and clinically actionable mutations [68], such as gastrointestinal stromal tumor (GIST) in about 80-85% of cases [69]. Although most c-*kit* alterations associated with cancer involve “gain-of-function” mutations that lead to constitutive activation of c-KIT in an SCF-independent manner, others entail amplification/overexpression or “loss-of-function” mutations [70].

Gain-of-function c-*kit* mutations have been found to represent oncogenic driver events in the development of a wide variety of cancers/proliferative diseases, including GISTs [71,72], some subtypes of melanomas [73], mastocytosis [74], acute myeloid leukemia (AML) [75], and seminomas [76].

In GIST, c-KIT expression is detected immunohistochemically in more than 95% of the cases. It has become an important diagnostic marker when used together with morphologic features displayed by these tumors [77,78]. In addition, 85%–90% of adult GISTs bear c-*kit* and *PDGFRA* gene gain-of-function mutations that are mutually exclusive [69] and seem unrelated to c-KIT expression, as they can be found in a proportion of GISTs that are immunohistochemically negative for c-KIT [77]. Mutations in c-*kit*, which in GISTs are more common than those in *PDGFRA*, most frequently involve the exon 11 that codes for the JM region, disrupting its autoinhibitory function and leading to constitutive activation of c-KIT [71,79]. Most of the mutations in exon 11 are caused by deletions and clusters between codons 550 and 560, which represents one of the hot spots within the c-*kit* gene [13,69]. Less common mutations occur in exon 9 that encodes the EC region of c-KIT, mainly involving an internal tandem duplication of Ala502-Tyr503 [71,80,81] that would mimic the conformational change that occurs when c-KIT dimerizes after binding its cognate ligand SCF [82]. Mutations also occur in exons 13 (encoding the ATP-binding region of c-KIT) and 17 (encoding the activation loop of the kinase), but are rare, with a combined frequency of 1-2% among all GISTs [81,83,84].

**FIGURE 3:**
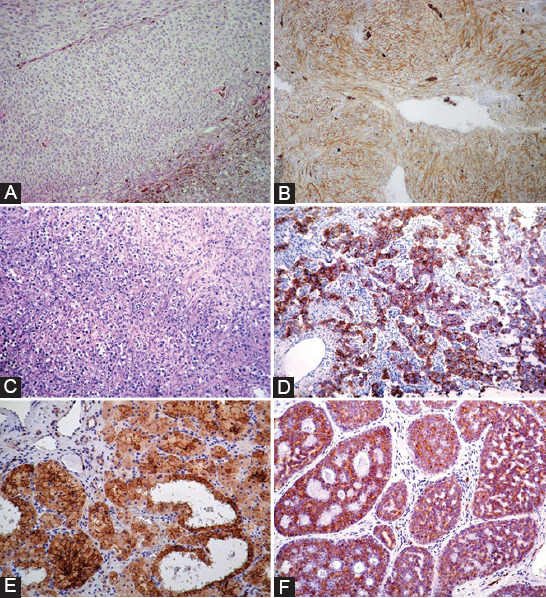
Examples of different cancer types expressing c-KIT. (A and B): Uveal melanoma morphology (A) with a strong and diffuse c-KIT expression; (C and D): Ovarian dysgerminoma (C) with C-KIT expression in cancer cells; tumor-infiltrating lymphocytes were negative (D); Renal oncocytoma (E) and mammary adenoid cystic carcinoma (F) exhibiting C-KIT positivity. All cases were stained immunohistochemically using polyclonal A-4502 antibody (DAKO Agilent). The images were taken at ×20 magnification except for C and D (×10 magnification).

In about 80% of melanomas, the main oncogenic drivers involve mutations in *B-Raf* proto-oncogene serine/threonine kinase (*BRAF*) and neuroblastoma rat sarcoma viral oncogene homolog (*NRAS*) (reviewed in [85]). However, c-*kit* mutations, which are typically mutually exclusive of *BRAF* and *NRAS* mutations, are identified in around 3% of melanomas, particularly those derived from acral surfaces (soles, palms, and nail beds) (36%), mucosa (39%), and chronically sun-damaged sites (28%) (reviewed in Reddy et al. [85], Pham et al. [86]). About 70% of mutations affecting c-*kit* in melanomas are constitutive activating mutations, including L576P (lysine-to-proline mutation at codon 576) in exon 11 and K642E (methionine-to-glutamic mutation at codon 642) in exon 13 [86]. Besides gene alterations, immunohistochemical studies have revealed overexpression of c-KIT in some melanoma variants, particularly among ocular melanomas (36-91%) [87-89]. In these cases, overexpression of c-KIT seems unrelated to c-*kit* mutations in exons 11, 13, 17, or 18 [88,89]. Moreover, c-KIT overexpression was associated with a worse outcome in patients with choroidal and ciliary body melanoma [90]. Figure 3A and B show representative immunohistochemical staining of a c-KIT-positive uveal melanoma.

Systemic mastocytosis is a rare myeloproliferative neoplasm in which malignant mast cells infiltrate bone marrow and other extracutaneous tissues such as liver, spleen, and peripheral blood. More than 90% of adult patients with this disease present a gain-of-function mutation in exon 17 within the c-*kit* gene, particularly KIT D816V, a missense mutation in which aspartic acid is substituted by valine in codon 816 [13,91]. Through kinase assays, it has been shown that the D816V mutant can autoactivate 586-fold faster than native c-KIT [79], which explains the adverse prognostic impact of the c-*kit* mutation in this hot spot in patients with systemic mastocytosis [91]. The D816V mutation is also present in children with systemic mastocytosis but with a lower frequency (42%). In contrast, other mutations occur in other locations, often in exons 8 and 9 (44%) that encode the fifth EC Ig-like domain [74], thus promoting a conformational change that enables dimerization and activation of c-KIT by lower physiologic SCF levels than normally needed [16].

c-KIT expression is seen in myeloblasts in 65-90% of AML patients [92,93] and, in some cases, co-expressed with SCF, suggesting a potential autocrine activation [13,92]. Furthermore, c-*kit* mutations are found in AML patients, predominantly associated with core-binding factor leukemias, an AML variant that involves chromosomal abnormalities t(8;21)(q22;q22) or inv(16)(p13q22)/t(16;16)(p13;q22) [94]. Most of these constitutive activating mutations mainly reside in exon 8 (as in-frame insertions or deletions that affect an EC domain involved in c-KIT dimerization) or exon 17 (as missense mutations that affect the activation loop in the c-KIT TK domain) [94].

Although an overall somatic mutation rate of about 8% has been reported in both seminoma and non-seminoma testicular germ cell tumors, the incidence of c-*kit* mutation is ten-fold higher (20-25%) in the former than in the latter [95,96]. The most common c-*kit* alteration in seminomas involves activating mutations in exon 17, mainly D816X (where X is either valine [V] or histidine [H]) [13,97]. Similar c-*kit* mutations have been reported in dysgerminomas (the ovarian counterpart of seminomas) [98,99], along with c-*kit* amplification associated with c-KIT protein overexpression evident through immunohistochemistry (IHC) (Figure 3C and D) [99]. Moreover, high expression of c-KIT in patients with primary ovarian high-grade serous carcinoma has been shown to be associated with shorter disease-free survival and peritoneal metastasis [100]. No correlation was found between c-*kit* mutations and c-KIT protein expression [99], which is detected in 78%–100% of ovarian dysgerminomas [101].

In addition to gain-of-function mutations described above for some cancers, different studies have shown overexpression of c-KIT in cancer cells that, in their normal cell counterparts, show very little or undetectable c-KIT expression when mainly assessed by IHC. For example, a 7-fold increase in c-*kit* mRNA expression relating to normal renal tissue has been reported in renal oncocytoma and chromophobe renal cell carcinoma (RCC) [102]. Moreover, IHC analysis performed in tissue microarrays (TMAs), including 226 renal tumors, revealed a strong c-KIT immunoreactivity in more than 85% of chromophobe RCCs and oncocytomas. In contrast, c-KIT expression was infrequently observed or undetectable in other renal tumors assessed [102,103]. IHC studies revealed c-KIT expression in 100% of cystic renal oncocytomas (Figure 3E) [104]. c-KIT overexpression in chromophobe RCC and renal oncocytoma was not associated with c-*kit* mutations [105].

Normal breast ducts and acini, but not myoepithelial and stromal cells, show some expression of c-KIT, which has been reported to be lost in most breast cancers [106-108]. This loss of c-KIT expression has been a potential determinant of malignant breast transformation due to c-*kit* gene promoter DNA hypermethylation [109]. Although a low percentage of breast carcinomas express c-KIT, if any, 20-42% of triple-­negative breast cancers, which lack expression of estrogen receptor, progesterone receptor, and HER-2/neu and have a significantly higher probability of relapse and poorer overall survival when compared with other breast cancer types, do express it [110-112]. Another type of breast cancer in which the expression of c-KIT is frequently seen is adenoid cystic carcinoma (ACC) of the breast that, although relatively clinically indolent, can be confounded with infiltrating duct carcinomas (particularly with tubular and cribriform carcinomas of the breast). In this context, IHC assessment of c-KIT is a valuable diagnostic tool since its expression is found in more than 90% of mammary ACC but not in other carcinomas with overlapping histologic features (Figure 3F) [113-115].

Although overexpression of c-KIT - rather than mutations in its gene - has been reported in a high percentage of small cell lung cancer (SCLC) patients by different groups [116-120], its prognostic relevance remains debatable due to conflicting findings that may be related to the type of tumor specimens used (biopsy or surgical samples), cancer stages, and other variables that still need to be scrutinized.

Expression of c-KIT and SCF has been reported in patient-derived immortalized colorectal cancer cell lines [121] and in premalignant and malignant colonic lesions, where c-KIT and SCF co-expression has been associated with a worse clinical outcome [122]. In a more recent study using a TMA comprising 137 patient-derived colon tumors and 179 associated serially passaged xenografts, it was found that c-KIT is expressed in approximately 50% of colorectal cancer tissues [123], in agreement with data collected from The Cancer Genome Atlas [124].

Several studies have assessed c-KIT expression in human prostate cancer (PCa) cell lines and biopsies taken from patients, though with some divergent results that may be due to the use of antibodies that have been discontinued some years ago and thus cannot be further employed for reproducibility analyses [125,126]. Studies carried out by our lab (RDB) using benign prostatic hyperplasia, primary tumors, and bone metastatic PCa specimens have shown uniform levels of SCF and a trend of increasing c-KIT expression that parallels disease aggressiveness [127]. These findings are in agreement with other studies that also revealed significantly increased expression of c-KIT in high-grade (Gleason score [GS] 8 or higher, or clinical Stage 2) compared with low-grade (GS 6-7, or clinical Stage 2) prostate tumors [128]. Despite this, we observed that most human PCa cell lines grown *in vitro* express c-*kit* at the gene level, though c-KIT immunoblotting only detects low or null protein levels [127], as shown similarly by others [129]. Using experimental models of bone metastasis, we observed *de novo* expression of c-KIT in intraosseous tumors generated by otherwise c-KIT-negative PCa cell lines, suggesting an induction of c-KIT expression in PCa cells by the bone microenvironment, which was confirmed by co-culture studies of PCa cells and bone marrow-derived cells [127,130]. Furthermore, we found that inhibition of bone-induced c-*kit* expression in PCa cells transduced with lentiviral short hairpin RNA could significantly reduce intraosseous tumor incidence and growth, suggesting a crucial role of this RTK in PCa bone metastasis [130].

## IMPLICATIONS OF C-KIT IN CANCER DEVELOPMENT AND PROGRESSION

A complex interplay of numerous biological processes contributes to the development and progression of cancer. In addition to studies reporting associations between specific gain-of-function or loss-of-function mutations in c-*kit*, induction of *de novo* expression or overexpression of c-KIT and clinical outcome in cancer patients (summarized above), research by many groups has revealed that c-KIT plays crucial roles in the regulation of many mechanisms leading to tumor formation and cancer progression in carcinomas. Below, we will describe some of these studies.

**FIGURE 4:**
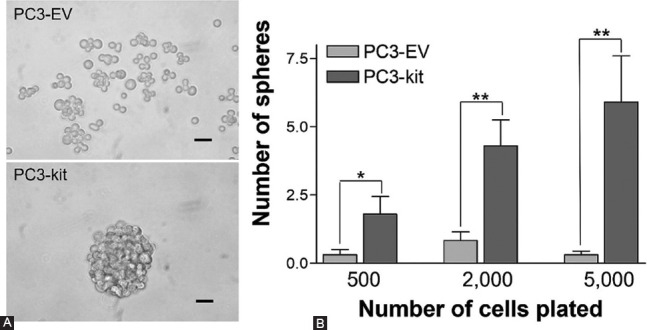
c-KIT expression and sphere formation in prostate cancer cells. (A) Representative image of prostaspheres formed by PC3 cells 6 days after transfection with c-*kit* and exposure to SCF under non-adherent 3D culture conditions (selective serum-free medium and non-adherent plates). Note that control PC3 cells transfected with the empty vector (EV) show little homotypic cell aggregation. Scale bars, 50 μm; (B) Quantitation of sphere formation by c-KIT-expressing and EV-expressing PC3 cells 6 days after plating at different numbers in non-adherent conditions. Data are expressed as the mean ± SE number of spheres larger than 50 μm per ten 100× microscopic fields. *p=0.05; **p<0.005 (Student’s test).

Cancer stemness, which refers to the cancer stem cell (CSC) phenotype, is characterized by the ability of a subpopulation of cancer cells to self-renew, differentiate into defined progenies, initiate tumor growth, and drive metastasis, recurrence, and resistance to therapies [131,132]. C-KIT has been proposed to regulate stemness in different cancers. Studies in ovarian cancer cells have related c-KIT expression to cancer stemness [133-136]. Accumulating evidence also indicates a role for c-KIT in colon cancer stemness, as supported by studies employing spheroid cultures derived from colon cancer patients’ tumor cells grown in serum-free and non-adherent plates, a technique commonly used to investigate CSCs [137]. These studies have shown that the release of SCF by more differentiated colon tumor cells modulates the growth of c-KIT-expressing CSC-like colon tumor cells [138], suggesting the existence of a paracrine system by which SCF can stimulate CSC-like cells found in the colonospheres. Furthermore, it was recently demonstrated that c-KIT stimulates CSC properties in colorectal cancer cells, including CD44 expression and other stem cell markers [139]. Studies on non-small lung cancer have also related c-KIT to cancer stemness, based on findings that revealed a reduction of CSCs through targeting the SCF-c-KIT autocrine signaling loop [140] and inhibition of c-*kit* with specific shRNA and inhibitors [141]. Moreover, in a recent study in which human PCa cell lines were separated into CD117 (c-KIT)-positive and CD117-negative cells, Kerr’s group demonstrated that in some instances c-KIT promoted sphere formation and increased the expression of specific stemness markers [142]. Similarly, we have observed that ectopic expression of c-KIT in PC3 cells followed by exposure to its ligand SCF increases the number of prostaspheres formed in selective serum-free medium and non-adherent plate conditions (Figure 4).

**FIGURE 5:**
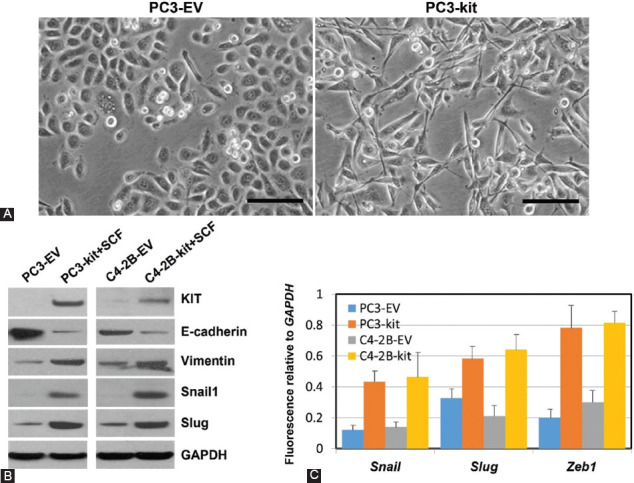
c-KIT expression and EMT-like phenomenon in prostate cancer. (A) PC3 cells stably transfected with c-*kit* show a fibroblast-like phenotype, while control PC3 cells transfected with the empty vector (EV) display the typical epithelial phenotype. Scale bars, 50 μm; (B) Western blots for c-KIT, epithelial-mesenchymal transition (EMT) markers, and EMT-related transcription factors are shown for PC3 and C4-2B PCa cells stably transfected with c-*kit* or EV and incubated with SCF (100 ng/mL for 60 min). Glyceraldehyde 3-phosphate dehydrogenase (GAPDH) served as a loading control; (C) Gene expression of *Snail*, *Slug*, and *Zeb1* is higher in c-KIT-expressing PC3 and C4-2B cells relative to EV-transfected control cells (RT-qPCR; *p*<0.001, Student’s test). Gene expression was normalized to *GAPDH*. Values are mean ± SE for triplicate samples.

To acquire migratory and invasive capacities, carcinoma cells must detach from adjacent epithelial cells and adopt a mesenchymal phenotype – the epithelial-mesenchymal transition (EMT), which plays a critical role in their aggressiveness and metastatic potential [143,144]. Regulators of EMT comprise different transcription factors such as Snail (including Snail and Slug, also called SNAI1 and SNAI2, respectively) and ZEB (Zeb1 and Zeb2), which can repress the expression of genes encoding E-cadherin and cytokeratins (associated with epithelial phenotype) and upregulate others encoding proteins linked to the mesenchymal phenotype (e.g., N-cadherin, vimentin, and fibronectin) [145-148]. Different studies have shown that the expression of EMT transcription factors is also increased in CSCs [147,149,150], and a growing body of evidence supports the view that circulating tumor cells (CTCs) can arise from tumor cells that have gone through EMT [151-154]. Acquisition of EMT properties and enhanced invasiveness and CSC traits has been found in salivary ACC cell lines after ectopic expression of c-KIT [155]. The association between KIT and EMT is also supported by immunohistochemical studies performed in a TMA comprising 150 specimens of thymic epithelial tumors, where the expression rate of c-KIT was found to be significantly higher in thymic carcinomas than in thymomas, which in most of the cases behave in a benign fashion and are noninvasive. In these studies, c-KIT expression positively correlated with EMT markers N-cadherin, Twist, and Snail and negatively with E-cadherin, suggesting that the immunohistochemical analysis of those proteins could be important to distinguish between thymic cancer and thymoma [156]. The association between c-KIT (CD117) and EMT is also supported by studies in ovarian cancer cells, where a reduction of CD117+ and CD44+ subpopulation of ovarian CSCs by metformin at low dose led to a significant decrease of Snail2, Twist, and vimentin related to mesenchymal traits, and an increase in expression of the epithelial marker E-cadherin [157]. In line with these findings, another group demonstrated that CD117+ subpopulations of human PCa cell lines present a significant increase in vimentin expression and *in vitro* migratory ability than CD117− subpopulations of the same cell lines [142]. In concordance with these results, we found that ectopic expression of c-KIT supports the *in vitro* migration and invasion of two different PCa cell lines along with BRCA2 downregulation, which may play a role in the process as suggested by gene rescue experiments [130]. We posit that the stimulation of the migratory and invasive abilities induced by c-KIT in these PCa cells would be mediated by an EMT-like phenomenon, as we observed a change in morphology toward a more mesenchymal phenotype, an increase in expression of Snail1, Slug, Zeb1, and vimentin, and a decrease in E-cadherin expression in c-*kit*-transfected PCa cell lines as compared to the same PCa cells transfected with the empty vector (EV) (c-*kit*-negative control cells) (Figure 5).

In addition to the contributory role exerted by cancer cell-intrinsic expression and activation of c-KIT in tumor development and progression, several lines of evidence suggest a key role for SCF-c-KIT signaling occurring in the tumor microenvironment. Perhaps the best example is that of mast cell infiltrates associated with tumors. Indeed, different studies in mice have demonstrated that high levels of c-KIT on mast cells and their presence in the tumor microenvironment promote angiogenesis, leading to increased tumor growth and metastasis [158-160]. Furthermore, additional research indicates that c-KIT and mast cells modulate the development, recruitment, and immunosuppressive effects of myeloid-derived suppressor cells in tumors [161,162].

## TARGETED THERAPY OF C-KIT-POSITIVE TUMORS

As previously outlined, various cancers present an aberrant activation of c-KIT kinase, caused either by overexpression or mutations in c-*kit*. Most of the 500 c-*kit* mutations identified so far in human cancer (Sanger Institute Catalogue of Somatic Mutations in Cancer [163]are passenger rather than driver mutations. To target and inhibit dysregulated c-KIT, two main approaches have been considered: small molecule inhibitors and monoclonal antibodies (mAbs). Among small molecule inhibitors, the first one developed was imatinib mesylate (Gleevec^®^), which was originally found to inhibit the TK activity of the chimeric BCR-ABL fusion oncoprotein resulting from the translocation t(9;22) in chronic myelogenous leukemia, and was approved for the treatment of this hematologic cancer in 2001 [164]. Serendipitously, imatinib was also found to inhibit the autophosphorylation and activation of some RTKs, such as c-KIT and PDGFR, and was approved as standard first-line treatment for metastatic GIST. It is also used in the adjuvant setting for patients with GISTs who may have potential curative treatment by surgery and for the treatment of adult patients following surgical removal of CD117-positive GISTs (reviewed in Kelly et al. [165]). Although imatinib can traverse the cell membrane and bind to JM and cytoplasmic enzymatic domains due to its small size (Molecular Weight: 493.6), its therapeutic effect is highly dependent on the mutation involved [70]. For instance, in GIST patients whose tumors harbor gain-of-function point mutations in the exon 11 JM domain of c-*kit*, found in 75-80% of the cases, imatinib provides a robust initial clinical response [71]. However, in almost 90% of these patients, there is a relapse of the disease within 20-24 months [166-169], which is due to secondary mutations in c-*kit* that usually cluster in exons 13/14 (the ATP-binding pocket) and 17 and 18 (the activation loop) of the kinase domain, preventing optimal binding of imatinib and restoring c-KIT signaling in the presence of the inhibitor (reviewed in Serrano et al. [169]). This has led to the approval of other TK inhibitors, such as sunitinib and regorafenib, with activity against secondary c-*kit* mutations [169]. Among these, there are agents such as sunitinib, which elicits longer progression-free survival and overall survival in patients that harbor exon 13 or 14 secondary c-*kit* mutations compared to those with exon 17 or 18 secondary c-*kit* mutations [167,168], and regorafenib, which has equal efficacy in tumors with secondary exon 13/14 or exon 17/18 mutations, or combinations thereof [170]. Ripretinib, a novel type II switch control kinase inhibitor, is a broad-spectrum inhibitor of secondary drug resistance mutations, including activation loop mutations targeted by type I inhibitors [171]. In addition, because PDGFRA-mutant GIST accounts for up to 10% of GISTs that exhibits primary resistance to imatinib and sunitinib therapy [165], other agents that selectively target *PDGFR*a D842V mutant advanced GISTs are of immense clinical value. Among them, we find avapritinib, an inhibitor of c-KIT and PDGFRA activation loop mutants that has been approved by the Food and Drug Administration (FDA) for GISTs that harbor *PDGFRA* exon 18 D842V mutations [167], whereas dasatinib, an oral inhibitor of c-KIT, produced a positive response in one patient with *PDGFRA* D842V-mutant GIST in a Phase II trial, and is currently used off label for this molecular subtype [172].

The clinical experience of imatinib in GIST led to studies to explore the potential therapeutic value of this TK inhibitor in systemic mastocytosis. In adult patients affected with the disease, the activating D816V c-*kit* mutation is found in about 90% of the cases and is responsible for primary resistance to imatinib. In contrast, in the remaining patients (with the absence of D816V c-*kit* mutation or unknown c-*kit* mutational status), a clinical response to imatinib was found, leading to its approval as a treatment by the FDA in 2006 [173-175]. This clearly demonstrates the relevance of identifying specific c-*kit* mutations to select patients for more adequate treatments.

To overcome the resistance developed in certain wild-type or mutant c-KIT-positive cancers treated with TK inhibitors such as imatinib, it has been proposed the use of mAbs to target and inhibit dysregulated c-KIT. Although unlike small molecule inhibitors, antibodies can only recognize EC epitopes of c-KIT, this may represent a potential therapeutic advantage due to their specific binding to both mutant and wild-type c-KIT receptors (recall that most c-kit mutations localize to JM or IC domains of the receptor). Using KIT-expressing NIH 3T3 and Ba/F3 cell lines, Shi et al. evaluated the feasibility of targeting oncogenic c-*kit* mutations using anti-D4 mAbs that obstruct homotypic D4 or D5 contact formation [176]. Oncogenic c-*kit* mutations were divided into two classes. Class I mutants include D5 point mutations D419A and N505I, deletion of Y418 D419, and duplication of A502Y503, which exhibit surface expression of constitutively activated TK activities. In contrast, Class II mutants, including the D5 T417ID418-419 mutation and the IC V560D and D816V point mutants, have constitutively activated TK activity with low or low negligible surface expression [176]. Anti-D4 mAbs abrogated oncogenic c-KIT signaling in mutations localized in D5, including all Class I mutants and the Class II T417ID418-419 mutation. Based on these findings, the authors proposed differential pharmacological treatment regimens for cancer patients depending on the c-*kit* mutations present in their tumors [176].

Moreover, antibody-drug conjugates (ADCs) can also be designed by conjugating different drugs to mAbs to deliver a potent cytotoxic payload to cancer cells while minimizing toxicity to normal tissue [177]. Studies with LOP628 [178] and NN2101-DM1 [179], two humanized anti-KIT antibodies conjugated to the tubulin polymerization inhibitor emtansine (DM1) [177], have been reported. Both ADCs showed strong *in vitro* antiproliferative activity on several c-KIT-positive human tumor cell lines representing GIST, AML, SCLC, and systemic mastocytosis regardless of their c-*kit* mutational status, and *in vivo* antitumor responses in imatinib-sensitive and -refractory GIST and systemic mastocytosis xenograft models, as well as in SCLC and AML models [178,179]. In both cases, the ADCs bind to c-KIT on the surface of the cancer cells, forming a complex that is then internalized and rapidly trafficked to the lysosome, releasing DM1 in the cytoplasm, arresting the cell cycle by inhibiting microtubule polymerization and leading to apoptosis of cancer cells models [178,179]. Despite the promising preclinical results obtained with LOP628, rapid hypersensitivity reactions were observed in some patients treated with this ADC in a Phase I clinical trial experienced, which led to a termination of the trial [180]. This unexpected outcome is likely caused by mast degranulation resulting from a high affinity binding of the Fc region of LOP628 to the Fc-gamma receptor on mast cells and an SCF-mediated c-KIT activation that is not inhibited by LOP628 (recall that c-KIT is expressed by mast cells) [180]. Although clinical studies are still needed to define the safety profile of NN2101-DM1, it has been demonstrated that the NN2101 antibody has decreased binding affinity to Fc receptors and an inhibitory action on SCF-dependent c-KIT activation [181], which might prevent hypersensitivity reactions such those observed with LOP628 (studies in patients are necessary to examine this hypothesis). Moreover, *in vivo* and *in vitro* studies revealed synergistic inhibitory effects on some cancer cells when treated with NN2101-DM1 and imatinib or carboplatin/etoposide [179]. This suggests that the use of combination therapies involving novel anti-KIT ADCs in conjunction with standard chemotherapeutic agents, TK inhibitors, or other targeted agents, should be considered a strategy to enhance the efficacy of anti-KIT ADCs used as a monotherapy for different cancer types.

## CONCLUSIONS

Knowledge of the contribution of SCF and c-KIT to different physiological mechanisms has increased dramatically during the last decades. Furthermore, accumulating evidence suggests that activating mutations or amplification/overexpression of c-*kit* contribute to the development and progression of many human malignancies, as supported by gene and protein profiling of clinical specimens and numerous *in vitro* and *in vivo* studies at elucidating the role played by c-KIT in cancer. Following the great success of imatinib in treating GISTs, other broad TK inhibitors have been approved to overcome the resistance acquired by certain c-KIT-positive tumors through secondary mutations occurring in c-*kit*. The identification of specific c-*kit* mutations could be of importance in recognizing more potent and selective treatments in certain c-KIT-positive tumors. However, the experience with TK inhibitors suggests an almost ever-present potential for the outgrowth of resistant cancer clones. Recent studies suggest that anti-KIT monoclonal ADCs may represent a new modality to treat wild-type and activating-mutant c-KIT-positive tumors, irrespective of their c-*kit* mutational status. The refinement of these highly selective therapeutic tools, either alone or combined with chemotherapeutic agents, TK inhibitors, or immune checkpoint inhibitors, will help treat cancer types driven by the c-KIT signaling machinery. These therapeutic strategies, if successful, hold the potential to significantly minimize toxicity to normal tissue and improve patient clinical outcomes.
